# Spectrum of childhood nephrotic syndrome in Iran: A single center study

**DOI:** 10.4103/0971-4065.73432

**Published:** 2010-10

**Authors:** S. A. Zaki, P. Shanbag

**Affiliations:** Department of Pediatrics, Lokmanya Tilak Municipal General Hospital, Sion, Mumbai - 400 022, India

Sir,

We read with interest the article by Safaei *et al*. on “Spectrum of childhood nephrotic syndrome in Iran: A single center study”.[1] We appreciate the work done by the authors. However, we have a few queries which we would like to be clarified.

The first indication for kidney biopsy mentioned in the study, i.e., age of onset between zero and 14 years will include all children in the study group. However, kidney biopsy was performed by the authors only in 17 children. We would like the authors to quote a reference supporting this indication for kidney biopsy. The Indian Pediatric Nephrology group and standard pediatric textbooks recommend that kidney biopsy in nephrotic syndrome should be done in children with age of onset <1 year or >8 years.[2,3]

The authors have also performed kidney biopsy in children with microscopic hematuria. Transient microscopic hematuria is found in 20–23% of steroid-sensitive nephrotic syndrome and usually does not warrant a kidney biopsy.[4] Various studies and standard textbooks recommend that kidney biopsy in nephrotic syndrome should be done in children with gross hematuria and/or persistent microscopic hematuria.[2–4]

The definition of steroid dependent nephrotic syndrome (SDNS) in the article is incorrect. As per the authors, SDNS is defined as recurrence of nephrosis when the dose of corticosteroids is reduced or within 2 months after the discontinuation of therapy. The reference quoted is from the 18th edition of Nelson Textbook of pediatrics. We would like to bring to the notice of the authors and our readers that the correct definition mentioned in the textbook is “recurrence of nephrosis when the dose of corticosteroids is reduced or within one month after the discontinuation of therapy”.[2]
